# Rival Systems of Ward Ventilation

**Published:** 1911-09-16

**Authors:** 


### HOSPITAL ARCHITECTURE AND CONSTRUCTION.

[Communications on this subject should be marked "Architecture" in the left-hand top corner of the envelope.]

### RIVAL SYSTEMS OF WARD VENTILATION.

The late Miss Florence Nightingale in her excellent work gives "Fresh Air" as the first essential to the health of the sick. The correct proportion of the ward in its three dimensions—height, length, and width—is an important factor in obtaining a continual movement of the atmosphere. Modern work is based generally on a height of 13 feet, a length of 10 feet centre to centre of beds, and a width of 27 feet. These dimensions give superficial area of 135 feet and cubic area of 1,755 feet per patient. Assuming that the natural ventilation obtained by windows, assisted in all probability by the fireplace or stoves, is sufficient to cause movement in the internal atmosphere, the patients will enjoy several changes of air per hour, but we are going on the hypothesis that the external atmosphere is sufficiently different from that inside the ward to cause this change. Sometimes we find the external atmosphere itself almost stagnant, and it is this state which gives rise to the question, Is it desirable by some means to cause movement so that the exhalations from the patients will be carried off? It is acknowledged that variations in temperature are necessary for maintaining healthy people in health, and we have no logical right to assume this natural law is different in cases of sickness.

Realisation of this truth will at once dispose of the method of ventilation which consists of pumping air at a fixed temperature into the wards at such a pressure that all openings are means of outlet rather than ingress. Such systems as the "Cyclone" of Messrs. Matthews and Yates, Ltd., are admirably adapted for industrial buildings, and in exceptional cases where a hospital is unfortuntely placed near bleach-works, mills, or chemical factories it might be expedient to use such a system based on filtered and tempered air. It must not be forgotten, however, that we are here dealing with a pavilion type of ward. In the ingenious plan of the Royal Victoria Hospital, Belfast (Mr. William Henman, F.R.I.B.A., architect), the seventeen wards are placed side by side and lighted by roof-lights. There are no cross-currents provided for, and the whole is said to be successfully ventilated on the Plenum principle. Plenum ventilation would be extravagant for the usual pavilion type of hospital, as there is so much length of corridor, and the various disconnections by cross-draughts would be fatal to the success of the system. The scheme of ventilation best suited to the pavilion ward is that best known as the Aspirating system, whereby the vitiated and heated air escapes through openings into ducts in or near the ceiling. These ducts by being heated in some manner cause an upward draught, and the efficiency of the scheme may be accelerated by an exhaust fan placed near the outlet of the collected ducts. One of the salient advantages of this system is that it permits flushing of the ward with air whenever desired. There is no external complicated mechanism to get out of order, gas burners being sufficient to cause the necessary upward draught.

Probably the most economical way to provide for ventilation of the ward on this system is by flues as large as the thickness of the walls will permit, lined with glazed fire-clay tubes, as might be used for linen shoots, so arranged that accumulated dust can be easily removed when the wards are cleaned. There is no doubt that the hospital ward of the future will be ozonised up to the standard of a gentle sea breeze, and this will probably revolutionise the pavilion system of planning while still ensuring to the patients a health-restoring atmosphere. The usual method of causing a movement of air now in vogue is by hot water heating on the low-pressure system, but the objection to this system is the large dust-accumulating areas made necessary by radiating coils and pipes. Much is to be said for the open fire at the end of a ward which looks so cheerful; the throat of the chimney should be well above the level of the beds so as not to cause draughts thereon. This may be attained by raising of the hearth some six to twelve inches. The chimney thus provided is the best of ventilating agents, it takes the air from the ward so successfully that, as has been proved by direct experiment, a single flue will in certain directions of the wind remove 60,000 cubic feet of air per hour, an inestimable acquisition to the atmosphere of the ward.

Central stoves in wards are popular, but they are a disfigurement and take up valuable floor space; they also warm the air where it is least required, and the horizontal flues rendered necessary are a source of trouble. Recent developments in consumptive buildings suggest that patients kept warm by bed coverings do not require heated air to breathe, and it would seem that in past days in General Hospitals too much has been made of heating the atmosphere and too little attention paid to keeping the patients comfortable by bed coverings.

